# In silico veritas? Potential limitations for SARS-CoV-2 vaccine development based on T-cell epitope prediction

**DOI:** 10.1371/journal.ppat.1008607

**Published:** 2020-06-04

**Authors:** Sandra Silva-Arrieta, Philip J. R. Goulder, Christian Brander

**Affiliations:** 1 IrsiCaixa AIDS Research Institute, Hospital Germans Trias i Pujol, Institute for Health Science Research Germans Trias i Pujol (IGTP), Badalona, Spain; 2 Department of Paediatrics, University of Oxford, Oxford, United Kingdom; 3 HIV Pathogenesis Programme, The Doris Duke Medical Research Institute, University of KwaZulu-Natal, Durban, South Africa; 4 Ragon Institute of MGH, MIT, and Harvard, Cambridge, Massachusetts, United States of America; 5 University of Vic–Central University of Catalonia, Catalonia, Vic, Spain; 6 Catalan Institution for Research and Advanced Studies (ICREA), Barcelona, Spain; University of Notre Dame, UNITED STATES

The development of a vaccine able to prevent infection or severe disease course of SARS-CoV-2 is a priority to stem the current COVID-19 pandemic and to be better prepared for future flare-ups. To accelerate T-cell immunogen design, many current approaches are employing epitope prediction strategies. Although such approaches have great merit, it is also important that unbiased approaches to characterizing the T-cell response to SARS-CoV-2 are incorporated into vaccine design, in order to generate a comprehensive picture of the total virus-specific T-cell response and to define correlates of protective immunity against the virus.

Ever since the first identification of binding motifs for T-cell antigens presented by HLA class I molecules by Rammensee and colleagues almost 30 years ago, epitope identification has been greatly facilitated by epitope prediction algorithms [1]. Over the years, many vaccines designs targeting infectious pathogens as well as cancer neoantigens have been based on in silico prediction of potential HLA class I–restricted epitopes, and a series of vaccine candidates that apply such strategies to SARS-CoV-2 are currently in development. However, even though numerous prediction algorithms have been developed and gradually improved, there are several considerations that may threaten or limit the success of such approaches.

## Epitope length is highly variable and not properly captured by prediction algorithms

Although the initial characterization of eluted HLA class I epitopes by the Rammensee lab in 1991 produced predominantly 9-mer epitopes, there was already the notion of longer and shorter sequences that can be bound and effectively presented by HLA class I molecules [1]. This observation has been validated many times over since, with some HLA class I alleles for which the 9-mer seems to be rather the exceptional length than the rule (such as HLA-B8, B35, B57, B27, and others). In fact, even the first identified epitopes in influenza and HIV infection, defined by other approaches a couple of years before the first binding motif was described, turned out to be 13 and 10 amino acids in length, respectively [2,3]. Especially for HIV, we have equally fallen victim to consider “optimal” HLA class I epitopes those for which functional experiments have identified the shortest reactive peptide and thereby unwillingly contributed to cementing the dogma of 9-mer optimal epitope length as the rule [4]. However, based on elution studies by many laboratories and crystallographic structural analyses, it is evident that many epitope variants presented by HLA class I molecules can exceed the 9-mer length [5,6]. In fact, even for members of the HLA-A11 allele family that have very strict and limited C-anchor binding requirements, elution studies have demonstrated that only about 20% of the (self and viral) epitopes are exactly 9 amino acids in length [7]. Of note, this was done in a system that uses soluble class I molecules secreted from the antigen-presenting cells and thus limits the bias to also identify (longer) processing intermediates. Similar observations have been made for other HLA class I alleles—for instance, HLA-B8, which consistently presents peptides that are shorter than 9-mers; or HLA-B57, for which the immunodominant and protective HIV-specific epitope is an 11-mer; or where epitopes have been found to be entirely embedded in other epitopes [8], all cases that are generally not identified or scored as independent epitopes by prediction algorithms.

## Multiple specificities toward the same epitope regions can be elicited by allowing for length variants

One major limitation of using predicted epitope sequences of 9 amino acids in T-cell vaccine designs is that T-cell responses to only that specific region will be induced by the vaccine. However, the example of HIV infection (which likely has the best-characterized epitope “landscape” described) shows how this can limit the vaccine-induced response: by (1) screening just 5 amino acids up- and downstream of each described optimal (mostly 9-mer) epitope for suitable anchor positions satisfying the currently known allele-specific binding motif and (2) allowing for an epitope length of 9 to 12 amino acids, the number of potential independent epitopes that could bind the given HLA class I molecule and induce T-cell responses to the same region more than doubles [5]. Although this may be of paramount importance to cope with HIV variability and to prevent rapid cytotoxic T-lymphocyte (CTL) escape, other viruses, even genetically robust DNA-based viruses such as Epstein-Barr virus (EBV), have been shown to present as swarms of quasi-species and to be able to develop effective CTL escape variants as well [9,10]. Thus, it seems critical that SARS-CoV-2 vaccine development take this aspect into account and test immunogen designs that can target the same epitopic region by multiple specificities, ideally composed of a polyclonal T-cell receptor repertoire to (1) broaden the response and thus increase the chances of eliciting more potent clonotypes and (2) more effectively prevent T-cell escape.

## HLA class I binding is promiscuous and motifs are poorly defined for less well-studied HLA class I alleles

There are to date more than 20,000 HLA class I alleles described that translate into more than 11,000 different HLA class A, B, and C molecules. For many of these alleles, sequence similarities and structural analyses have allowed grouping them into larger, so-called HLA supertypes that share epitope binding similarities [11]. However, there is extensive binding promiscuity even by short 9-mer peptides that go well beyond the specific allele and its assigned supertype and for which commonly used minimal cutoffs of binding affinities would not identify the reactive peptides [12]. In fact, screening data using several hundred epitopes derived from different viral infections suggest that the current prediction algorithm may miss a large number of reactive “optimal” epitopes and possibly an even larger proportion of responses when predicting potential epitopes on a full protein sequence [12]. An extreme example of this may be epitopes predicted (and presented) by HLA-E, for which binding peptides showed an unexpected broad permissiveness at anchor positions and extensive structural freedom to bind to the presenting class I molecule [13]. The current urgency by which the scientific community attempts to achieve a viable T-cell vaccine to SARS-CoV-2 will, however, not allow generation of larger training sets that could improve these predictions and thus may mislead the design into regions of the virus that are rich in epitopes fulfilling binding motifs of the most commonly studied class I alleles. In addition, these approaches will not be able to sufficiently cover the genetically different, and often more numerous, populations with less well-characterized genetics and, in parallel, less well-established healthcare systems in which vaccines may be the only way to stem against the pandemic.

## Antigen-processing preferences and TAP-mediated peptide translocation

Another critical limitation that is not properly addressed by most epitope prediction tools is the need to identify appropriate antigen-processing sites that could give rise to predicted, presented epitopes. As the composition of the antigen-processing machinery changes upon proinflammatory signals and can differ between different cell types, currently known prediction algorithms will likely not be suited to estimate the processing efficacy of viral antigens in the context of an infected target cell. Thus, without functional validation, there exists the risk that predicted epitopes may represent great theoretical or actual binder to HLA class I molecules, but they are either not processed at all or not processed at sufficient levels to induce a strong immune response and to sensitize infected target cells for CTL-mediated killing. In addition, the allelic diversity of TAP (transporter associated with antigen processing) genes, which encode for proteins that form a bottleneck for the translocation of processed antigen into the lumen of the endoplasmatic reticulum, are only partly integrated into peptide prediction algorithms.

## Functional characterization, cross-reactivity, and immunopathology

Finally, there are evident advantages in identifying antigens not only to protect from SARS-CoV-2 infection but to develop vaccine candidates that could act against past and future coronavirus outbreaks. This would call for focus on particularly conserved regions between isolates and different coronaviruses, something that can be readily implemented in epitope prediction strategies, even if conservation on an epitope level does not need to be complete. Whether such cross-reactive T-cell responses could indeed mediate cross-protection and are associated with effective control of different coronaviruses, remains to be seen. Of note, emerging data start providing some insights into T-cell effector function profiles that may be protective and those that may be associated with a more severe clinical course of SARS-CoV-2 infection [14]. This is reminiscent of the situation in Dengue virus infection, in which some cross-reactive T-cell responses have been associated with the observed immunopathological consequence [15]. Thus, a functional assessment of T-cell responses in individuals with mild, moderate, and severe COVID-19 disease courses may be indicated, so that potential detrimental effects of preexisting, cross-reactive T-cell immunity can be avoided. However, without an unbiased screening for total virus-specific T-cell responses with classical and alternative T-cell effector functions [16], it will be difficult to differentiate most beneficial from potentially harmful T-cell specificities.

## Conclusions

With the urgency to develop effective measures to control the COVID-19 pandemic and to design vaccine strategies useful to confront future outbreaks and epidemics of SARS-CoV-2 and related coronaviruses, accelerated programs for T-cell immunogen design are needed. T-cell epitope prediction algorithms are an effective tool to narrow down the potential immunogen cargo in a future SARS-CoV-2 vaccine from the total viral proteome. However, approaches exclusively based on 9-mer epitope prediction will potentially miss critically important responses, and even those based on 9-mer and 10-mer epitope prediction have similar shortcomings, for the reasons described above. In addition, a relatively unbiased approach to characterizing the T-cell response using overlapping peptides will facilitate understanding of immune correlates of SARS-CoV-2 control versus disease. The benefit of the predicted 9-mer/10-mer approach would be that, when used in combination with panels of overlapping peptides spanning the viral proteome, optimal epitopes will be more rapidly identified and, at the same time, immune correlates of disease protection evaluated in an unbiased fashion. These considerations should not be overlooked, as invaluable time and resources could be directed in directions that may not yield the desired success.
